# Increased frequency of Th17 cells in systemic sclerosis is related to disease activity and collagen overproduction

**DOI:** 10.1186/ar4430

**Published:** 2014-01-07

**Authors:** Xiaoqin Yang, Ji Yang, Xiaojing Xing, Linlin Wan, Ming Li

**Affiliations:** 1 Department of Dermatology, Zhongshan Hospital, Fudan University, 180 Fenglin Road, Shanghai 200032, China; 2 Department of Dermatology, Shanghai Skin Diseases Hospital, 200 Wuyi Road, Shanghai 200050, China

## Abstract

**Introduction:**

Although immune dysfunction plays a role in the pathogenesis of systemic sclerosis (SSc), involvement of T helper 17 (Th17) and T regulatory (Treg) cells remains unclear. We aimed to investigate the presence of Th17 and Treg cells in SSc patients and the role of Th17 cells in collagen production in SSc fibroblasts.

**Methods:**

We analyzed inflammatory cell profiles in the skin of 13 SSc patients by immunohistochemistry, the percentage of Th17 and Treg cells in peripheral blood mononuclear cells (PBMCs) of 45 SSc patients and 24 healthy controls by flow cytometry, gene expression in PBMCs by real-time reverse transcription-polymerase chain reaction and interleukin-17 (IL-17) in sera and culture supernatants by enzyme-linked immunosorbent assay. We also investigated the effect of Th17 cell-derived *IL-17* on fibroblast growth and collagen production.

**Results:**

Infiltration of inflammatory cells including IL-17^+^ and Foxp3^+^ lymphocytes was detected in the skin of patients with early SSc. The percentages of circulating Th17 cells and *IL-17* production were elevated in samples from patients with active SSc, whereas the percentage of circulating Treg cells was not affected. The number of Th17 cells was closely related to disease activity. *IL-17* from SSc patients promoted fibroblast growth and collagen production, whereas *IL-17* neutralizing antibody effectively blocked collagen production.

**Conclusion:**

SSc progression might be linked to expansion of circulating Th17 cells and increased infiltration of IL-17^+^ cells in skin. Th17-derived *IL-17* is involved in fibroblast growth and collagen production. *IL-17* blocking antibody may be a useful tool for intervention in the fibrotic course of SSc.

## Introduction

Systemic sclerosis (SSc) is a complex inflammatory autoimmune disease characterized by excessive deposition of collagen that leads to fibrosis of multiple organs, including the skin, lungs, heart, and gastrointestinal tract, and is often associated with widespread vasculopathy and immunologic abnormalities [1].

A unique feature of SSc that distinguishes it from other fibrotic disorders is that autoimmunity and vasculopathy characteristically precede fibrosis. Although immunomodulatory drugs have been used extensively in the treatment of SSc, to date, no therapy has been able to reverse the progression of tissue fibrosis or substantially to modify the natural progression of the disease. This is mainly because the mechanisms responsible for the initiation and progression of the disease have not been clearly identified.

Growing evidence suggests that T-cell proliferation and cytokine secretion play a major role in the pathogenesis of SSc [2-4], suggesting that this condition could be associated with a general defect in the control of T-cell activation [3]. Recently, a subset of T-helper cells was described and named T helper 17 (Th17) cells, based on their production of interleukin (IL)-17A, IL-17F, and IL-22 [5,6]. *IL-17* concentration was reported to be elevated in the serum of SSc patients [7,8]. This finding was further confirmed in more recent studies, which reported drastically increased proportions of Th17 cells in SSc patients [9-11]. Our previous study showed that Th17 cells are expanded in systemic lupus erythematosus (SLE) patients, and Th17 cell-derived *IL-17* is related to recruitment of inflammatory cells to vascular endothelial cells [12]; however, the role of Th17 cells and *IL-17* in the fibrosis of SSc is not clear. Naturally occurring CD4 regulatory T (Treg) cells maintain immune balance and control the inflammatory injuries [13,14]. It has been suggested that Th17 and Treg cells are produced in a reciprocal manner, depending on the levels of potentially proinflammatory or antiinflammatory cytokines and activation of specific transcription factors [15,16]. Thus, we hypothesized that altered cytokine profiles in SSc patients might result in an imbalance of Th17/Treg cells, and might be responsible for the prominent features of SSc, such as fibroblast proliferation and endothelium injury [2,17].

Here, we first demonstrated increased IL-17^+^ and Foxp3^+^ lymphocyte infiltration in the lesions of patients with early SSc. In detailed studies of circulating Th17 and Treg cells in 45 SSc patients, we showed that Th17 cells exhibited global expansion in peripheral blood rather than redistribution *in vivo*, and this expansion of Th17 cells was related to disease activity but was not correlated with Treg cell depletion during disease flare. Further studies demonstrated that *IL-17* derived from patients with active SSc promoted fibroblast growth and collagen production, and neutralization of *IL-17* could alleviate the production of collagen. These data suggest that the pathophysiology of SSc might be linked to the expansion of Th17 cells, and that Th17-derived *IL-17* may play a key role in the fibrotic course of SSc.

## Methods

### SSc patients and healthy controls

This study was approved by the Ethical Committee of Zhongshan Hospital, Fudan University (Shanghai, People’s Republic of China). All SSc patients were referred to the Department of Dermatology at Zhongshan Hospital and all provided informed consent. Forty-five consecutive adult patients (36 women and nine men, mean age 50.9 ± 7.2 years) who met the American College of Rheumatology criteria for the classification of SSc were included in the study [18]. Among these, 20 patients were classified as having limited cutaneous SSc (lSSc), and 25, as having diffuse cutaneous SSc (dSSc), according to the system proposed by Le Roy *et al*. [19]. Disease activity was assessed by using the criteria proposed by Valentini *et al*. [20], in which evaluation of clinical and laboratory factors results in a score ranging from 0 to 10 (0 represents no disease activity, and 10 represents maximal activity) [20]. Thirteen patients (nine women and four men, mean age, 48.4 ± 7.2 years) with a score ≥3 were classified as the active SSc group. The stable SSc group comprised 32 patients (27 women and five men, mean age, 51.9 ± 14.2 years) with score a <3. SSc patients were treated with prednisone, or cyclophosphamide + prednisone.

For the control group, 24 age- and sex-matched healthy individuals (18 women and six men, mean age, 50 ± 9.2 years) were enrolled after providing informed consent.

For histochemistry analysis, skin tissue was obtained from skin biopsies of 13 SSc patients (the location of skin biopsies is the flexure side of the forearm). Disease stage was defined as proposed by Steen and Medsger: early lSSc, disease duration <5 years; late lSSc, disease duration ≥5 years; early dSSc, disease duration <3 years; late dSSc, disease duration ≥3 years; disease duration was from first non-Raynaud symptoms [21,22]. Eight patients were classified as early SSc (12 ± 7.0 months), five as late SSc (45 ± 8.8 months), 12 were classified as dSSc, and one as lSSc. Four age- and sex-matched healthy tissue samples were obtained with informed consent (three skin biopsies from healthy donors and one tissue from orthopedic surgery).

### Immunohistochemistry

Tissues were processed and embedded in paraffin by using routine methods. Tissue blocks were serially sectioned to obtain consecutive levels. Sections were stained with hematoxylin and eosin, and immunohistochemistry was performed as previously described [12] by using antibodies to CD3, CD4, CD8, CD20, CD68, *IL-17*, and *Foxp3* (all from Abcam, Cambridge, MA, USA). Immunohistochemical staining was assessed by two independent pathologists without knowledge of patient characteristics. The positive cells in per surface were counted under × 400 magnification, and five randomly selected independent microscopic fields were counted for each sample to ensure that the data were representative and homogeneous.

### Flow cytometry

For detection of Treg cells, PBMCs were stained with fluorescein isothiocyanate (FITC)-conjugated anti-CD4, phycoerythrin (PE)-Cy5-conjugated anti-CD25, and PE-conjugated anti-CD127 (BD Pharmingen, San Jose, CA, USA) according to the manufacturer’s protocol. We gated first on CD4^+^ T cells and then on CD25^+^CD127^-^ Treg cells, as previously described [12]. After staining, cells were washed twice and resuspended in FACS solution (phosphate-buffered saline (PBS) with 0.5% bovine serum albumin and 0.02% sodium azide), fixed in PBS containing 1% paraformaldehyde, and analyzed the same day in a FACS-Calibur (BD-Bioscience) followed by analysis with FlowJo (Tree Star).

For detection of Th17 cells, PBMCs were incubated for 4 to 5 hours with 50 ng/ml phorbol 12-myristate 13-acetate (PMA) and 750 ng/ml ionomycin (PI) in the presence of 20 μg/ml Brefeldin A (Sigma-Aldrich, St. Louis, MO, USA) in a tissue-culture incubator at 37°C. Surface staining with PE-Cy5-conjugated anti-CD3 and FITC-conjugated anti-CD8 (BD Pharmingen) was performed for 15 minutes, followed by resuspension in Fixation/Permeabilization solution (Invitrogen, San Diego, CA, USA), according to the manufacturer’s instructions. Intracellular staining of PE-conjugated anti-IL-17 or isotype control was performed according to the manufacturer’s protocol (eBioscience, San Diego, CA, USA). For detection of Th17 cells, we first gated on CD3^+^ T cells, and analyzed CD8^-^IL-17^+^ T cells in a CD3^+^ gate, as previously described [12].

### Fibroblast isolation, culture, and stimulation

Fibroblasts producing high levels of collagen were isolated from the skin of SSc patients according to our previous modified limiting-dilution method [23]. Isolated fibroblasts were cultured in the presence of 20 ng/ml *IL-17* (eBioscience) for the indicated number of days, and the growth of fibroblasts was analyzed by 3-(4,5-dimethylthiazol-2-yl)-2, 5-diphenyltetrazolium bromide (MTT) assay. For gene-expression experiments, fibroblasts were cultured in different doses of *IL-17* for 48 hours, and *collagen 1* and *collagen 3* gene expression was analyzed by real-time reverse transcription-polymerase chain reaction (RT-PCR).

To determine the effect of secreted *IL-17* on collagen production, PBMCs from patients with active SSc were incubated for 4 to 5 hours with PI (Sigma-Aldrich), and supernatants were collected for later use. Fibroblasts isolated from the skin of SSc patients were cultured for 48 hours, and the culture media was replaced with Dulbecco modified Eagle medium (DMEM; Hyclone, Logan, UT, USA) containing 20% supernatant from the stimulated active SSc PBMC culture, and the cultures were incubated for a further 48 hours. Antibody to *IL-17* (eBioscience) was added to some cultures to a final concentration of 20 μg/ml. Culture media with the same doses of PI was used as a vehicle control. Collagen gene expression in fibroblasts was analyzed with real-time RT-PCR, and collagen secretion was analyzed by enzyme-linked immunosorbent assay (ELISA).

In similar experiments, isolated CD4^+^CD161^+^CD196^+^ Th17 cells [24,25] (eBioscience) were incubated for 4 to 5 hours with PI, and the supernatants were collected. Fibroblasts were cultured for 48 hours, and the culture media was replaced with DMEM (Hyclone) containing 20% supernatant from Th17 cells (4 × 10^5^ cells) from patients with active SSc or healthy controls. Antibody to *IL-17* was added to some cultures to a final concentration of 20 μg/ml. After incubation for another 48 hours, collagen secretion was analyzed with ELISA.

### ELISA

Sera were collected from SSc patients and healthy controls and frozen at -80°C until needed. Serum concentrations of *IL-17* were determined with ELISA (R&D, Minneapolis, MN, USA). In some experiments, isolated PBMCs were cultured and stimulated with PI (Sigma-Aldrich) for 5 hours before measurement of *IL-17* in the supernatants.

### Analysis of cytokine and transcription factor mRNA expression

Total RNA was purified with Trizol reagent (Invitrogen). cDNAs were synthesized by using ReverTra Ace-α- Kit (Toyobo), and mRNA expression was determined by using a SYBR green kit (Toyobo). The 2^-ΔΔCt^ method was used to normalize transcription to β-actin and to calculate the fold induction relative to controls. The following primer pairs were used: Hum *18S*, forward GCCCGAAGCGTTTACTTTGA and reverse TCCATTATTCCTAGCTGCGGTATC; Hum *Foxp3*, forward GAAACAGCACATTCCCAGAGTTC and reverse ATGGCCCAGCGGATGAG; Hum retinoic acid-related orphan receptor-gamma t (*RORγt*), forward TGAGAAGGACAGGGAGCCAA and reverse CCACAGATTTTGCAAGGGATCA; Hum *IL-17*, forward CAACCGATCCACCTCACCTT and reverse GGCACTTTGCCTCCCAGAT; Hum *COL1,* forward GTTGTGCGATGACGTGATCTGTGA and reverse TTCTTGGTCGGTGGGTGACTCTG; Hum *COL3,* forward CGGGTGAGAAAGGTGAAGGAGG and reverseAGGACCAGGAAGACCACGAGCA.

### Statistical analyses

Results were expressed as mean ± standard deviation. Statistical significance was determined by analysis of variance for comparisons of multiple means followed by the Bonferroni *post hoc* test or the Student *t* test and the Mann-Whitney *U* test. Correlations were determined with Spearman ranking.

## Results

### Inflammatory cell profiles in skin of SSc patients

**Figure 1:**
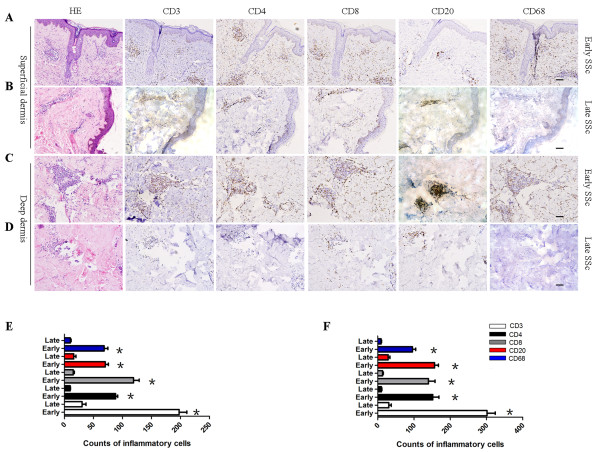
**The profile of inflammatory cells in the skin of SSc patients. (A)** Hematoxylin and eosin (H&E) staining of consecutive serial sections showing typical pathologic changes of SSc (left panel); lymphocyte infiltration confirmed by CD3, CD4, CD8, CD20, and CD68 immunohistochemical staining in superficial dermis of patients with early SSc (right panels). **(B)** H&E staining (Left panel); lymphocyte infiltration confirmed by CD3, CD4, CD8, CD20, and CD68 immunohistochemical staining in superficial dermis of late SSc patients (right panels). **(C)** H&E staining (left panel); lymphocyte infiltration confirmed by CD3, CD4, CD8, CD20, and CD68 immunohistochemical staining in deep dermis of early SSc patients (right panels). **(D)** H&E staining (left panel); lymphocyte infiltration confirmed by CD3, CD4, CD8, CD20, and CD68 immunohistochemical staining in deep dermis of patients with late SSc (right panels). **(E)** Counts of CD3^+^, CD4^+^, CD8^+^, CD20^+^, and CD68^+^ lymphocytes in superficial dermis of skin (early SSc patients, *n* = 8; late SSc patients *n* = 5). **(F)** Counts of CD3^+^, CD4^+^, CD8^+^, CD20^+^, and CD68^+^ lymphocytes in deep dermis of skin (early SSc patients, *n* = 8; late SSc patients, *n* = 5). The positive cells in the surface were counted under × 400 magnification, and five randomly selected independent microscopic fields were counted for each sample to ensure that the data were representative and homogeneous. Scale bar, 100 μm.

Previous histologic analysis of skin from SSc patients showed small pericapillary lymphocytic infiltrates [3]; however, it is not clear whether a specific immune response signature of the skin microenvironment occurs in SSc or whether the skin inflammation is governed by a predominantly immune response. In this study, among the 13 SSc patients enrolled, eight were classified as early SSc (12 ± 7.0 months), and five, as late SSc (45 ± 8.8 months). CD3^+^, CD4^+^, CD8^+^, CD20^+^, and CD68^+^ cells were examined with immunohistochemical staining of consecutive serial sections. Our data showed complex inflammatory cell infiltration but no predominant subsets of inflammatory cells. CD3^+^, CD4^+^, CD8^+^, and CD68^+^ cells were detected in both superficial and deep dermis of involved skin from patients with early SSc, with CD20^+^ cells mainly infiltrating pericapillary regions in the deep dermis (Figure 1A, B). The number of infiltrated cells was significantly decreased in skin from late SSc patients compared with early SSc (Figure 1C through F).

These data indicate that complex inflammatory cell infiltration is involved in the course of early SSc and that the inflammation reaction decreases in later stages of disease.

### Increased infiltration of IL-17^+^ and Foxp3^+^ lymphocytes in the skin of patients with early SSc

**Figure 2:**
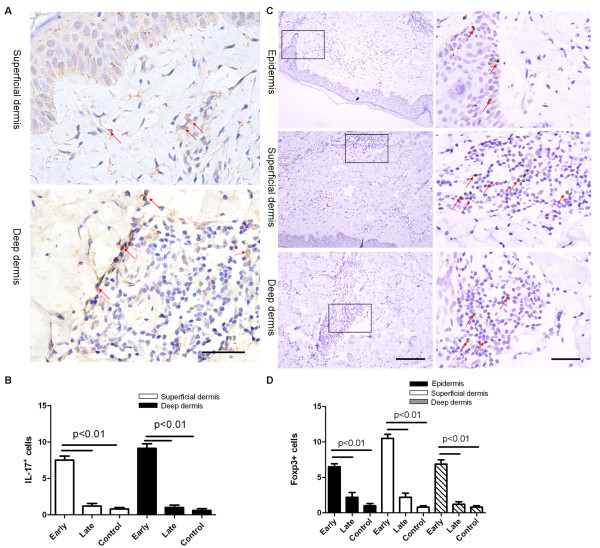
**Increased infiltration of IL-17**^**+ **^**and Foxp3**^**+ **^**cells in the skin of patients with active SSc. (A)***IL-17* protein expression in superficial dermis and deep dermis of early SSc patients examined by immunohistochemical staining. **(B)** Counts of IL-17^+^ lymphocytes in skin of SSc patients (early SSc patients, *n* = 8; late SSc patients, *n* = 5). The positive cells in surface were counted under × 400 magnification, and five randomly selected independent microscopic fields were counted for each sample to ensure that the data were representative and homogeneous. **(C)***Foxp3* protein expression in epidermis, superficial dermis, and deep dermis of patients with early SSc examined with immunohistochemical staining (left panels). Further magnification of the black-bordered box shows typical Foxp3^+^ lymphocytes (right panels). **(D)** Counts of Foxp3^+^ lymphocytes (×400 magnification) in skin of SSc patients (early SSc patients, *n* = 8; late SSc patients, *n* = 5). The positive cells in surface were counted under × 400 magnification, and five randomly selected independent microscopic fields were counted for each sample to ensure that the data were representative and homogeneous. Scale bar, 100 μm.

We analyzed the infiltration of IL-17^+^ and Foxp3^+^ cells in skin biopsy specimens from patients with SSc and healthy controls by using immunohistochemistry. The infiltration of IL-17^+^ cells, expressed as the number of cells showing superficial and deep dermal infiltration under × 400 magnification, was significantly increased in skin from lesions of early SSc patients (superficial dermis: 7.5 ± 1.6 cells; deep dermis: 9.1 ± 1.8 cells) compared with late SSc patients (superficial dermis: 1.2 ± 0.8 cells; deep dermis: 1.0 ± 0.7 cells, *P* < 0.01) and healthy controls (superficial dermis: 0.8 ± 0.4 cells; deep dermis: 0.6 ± 0.5 cells, *P* < 0.01, Figure 2A, B). The infiltration of Foxp3^+^ cells in the epidermis of lesional skin of early SSc patients (6.5 ± 1.2 cells) was significantly greater than the number observed in skin from late SSc patients (2.2 ± 1.5 cells, *P* < 0.01) and healthy controls (1.0 ± 0.7 cells, *P* < 0.01; Figure 2C, D). The infiltration of Foxp3^+^ cells in the superficial and deep dermis of early SSc patients (superficial dermis: 10.5 ± 1.6 cells; deep dermis: 6.9 ± 1.7 cells) was significantly higher than that in patients with late SSc (superficial dermis: 2.2 ± 1.3 cells; deep dermis: 1.2 ± 0.8 cells, *P* < 0.01) and healthy controls (superficial dermis: 0.8 ± 0.4 cells; deep dermis: 0.8 ± 0.4 cells, *P* < 0.01). These data suggest that both IL-17^+^ and Foxp3^+^ lymphocytes might be involved in the inflammation course of early SSc.

### The percentage of Th17 cells is expanded in SSc patients, but the percentage of Treg cells is not significantly affected

**Figure 3:**
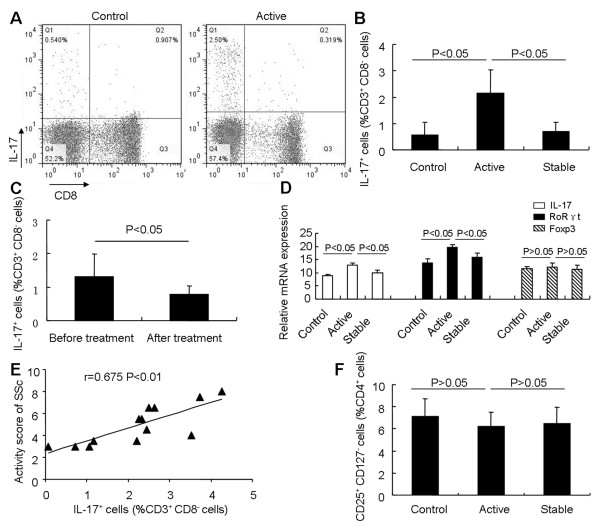
**Expansion of Th17 cells in PBMCs of patients with active SSc. (A)** Human PBMCs were labeled with antibody against lymphocytes (anti-CD3 and -CD8). IL-17-expressing cells were detected by intracellular cytokine staining assay, and the percentage of IL-17^+^ cells among CD3^+^CD8^-^ T cells was determined with flow cytometry. **(B)** Results of flow-cytometric analysis of Th17 cells in patients with active SSc (*n* = 13), patients with stable SSc (*n* = 32), and controls (*n* = 24). **(C)** Longitudinal monitoring of Th17 cells in 10 patients. The percentage of Th17 cells was measured initially during active SSc and again after resolution after treatment. **(D)** Real-time RT-PCR analysis of *IL-17, Foxp3, and RORγt* mRNA expression in freshly isolated PBMCs of patients with active SSc (*n* = 13), patients with stable SSc (n = 32), and controls (n = 24). **(E)** Positive correlation between the proportion of Th17 cells and clinical severity in active SSc patients, scored by using the Valentini score (*n* = 13). *r* = 0.675, *P* < 0.01. **(F)** Results of flow cytometric analysis of CD4^+^CD25^+^CD127^-^ Treg cells in patients with active SSc (*n* = 13), patients with stable SSc (*n* = 32), and controls (*n* = 24).

To investigate further these lymphocyte subgroups in PBMCs of SSc patients, we studied 45 patients with SSc, including 13 patients with active SSc and 32 with stable SSc. Twenty-four age- and sex-matched healthy individuals were also included. The percentage of circulating CD3^+^CD8^-^IL-17^+^ Th17 cells measured with flow cytometry (Figure 3A) was significantly increased in patients with active SSc (2.14 ± 0.89%, *n* = 13) compared with those with stable SSc (0.7 ± 0.34%, *n* = 32) and healthy controls (0.57 ± 0.49%, *n* = 24; Figure 3B). We next questioned whether the percentage of Th17 cells within the same individual varied in relation to disease status. Ten individuals who were tested longitudinally showed a decrease in the percentage of Th17 cells after treatment (1.31% ± 0.67% before treatment versus 0.78% ± 0.25% after treatment, *P* < 0.05, Figure 3C). *IL-17* is a key Th17-derived cytokine that promotes the inflammatory responses [26], and *RORγt* is a transcription factor that is expressed in Th17 cells [27]. Both of these genes were highly expressed in samples from patients with active SSc compared with samples from healthy individuals and patients with stable SSc (Figure 3D). Furthermore, comparison of the percentage of Th17 cells with respect to disease activity revealed a positive correlation between the percentage of Th17 cells and SSc activity characterized by Valentini score (*R* = 0.675; *P* < 0.01; Figure 3E). These results imply that Th17 cells might be involved in the SSc disease process.

Treg cells play a key role in peripheral immune tolerance and prevent the occurrence of autoimmune disease [13,14]. In this study, Treg cells were quantified by CD4^+^CD25^+^CD127^-^ T cells [12]. The percentage of CD4^+^CD25^+^CD127^-^ T cells decreased slightly, but not significantly, in patients with active SSc (6.25 ± 1.22%, *n* = 13) compared with patients with stable disease (6.47 ± 1.49%, *P* > 0.05, *n* = 32) and healthy controls (7.14 ± 1.61%, *P* > 0.05, *n* = 24; Figure 3F). The percentage of Treg cells was not related to disease activity and the expansion of Th17 cells in patients with active SSc (data not shown). Expression of *Foxp3*, a transcription factor in Treg cells, was not significantly different in patients with active SSc compared with patients with stable disease and healthy controls (Figure 3D).

### Th17-derived ***IL-17*** contributes to fibroblast proliferation and collagen production

**Figure 4:**
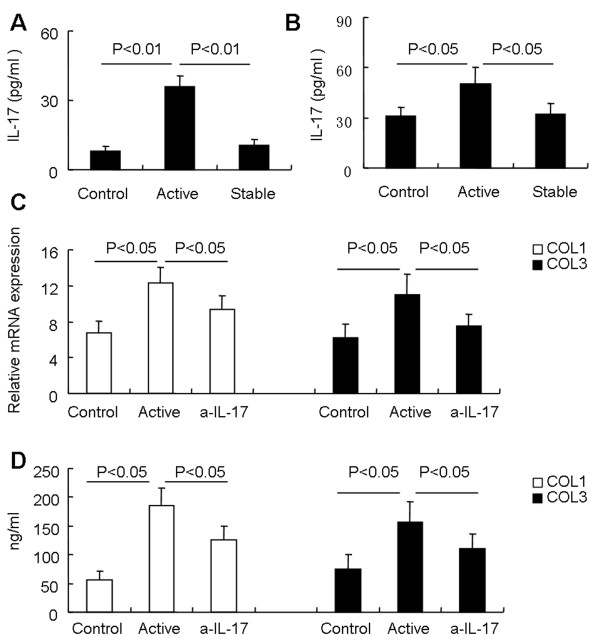
***IL-17* derived from SSc patients induces collagen production in fibroblasts. (A)***IL-17* concentration in sera of patients with active SSc (*n* = 13), patients with stable SSc (*n* = 32), and healthy controls (*n* = 24). **(B)** PBMCs from patients with active SSc (*n* = 6), patients with stable SSc (*n* = 6), and healthy controls (*n* = 6) were stimulated for 5 hours with PMA and ionomycin, and *IL-17* in the supernatant was measured with ELISA. **(C)** Fibroblasts were stimulated for 48 hours with supernatants from cultures of PI-stimulated PBMCs from healthy controls (Control) or, supernatants from cultures of PI-stimulated PBMCs from patients with active SSc (Active), and neutralization of *IL-17* (α-IL-17), and the gene expression of *collagen 1* and *collagen 3* was measured by real-time RT-PCR analysis. Results shown are representative of at least three independent experiments. **(D)** Fibroblasts were stimulated for 48 hours with supernatants from cultures of PI-stimulated PBMCs from healthy controls (Control) or, supernatants from cultures of PI-stimulated PBMCs from patients with active SSc (Active), and neutralization of *IL-17* (α-IL-17), and the levels of *collagen 1* and *collagen 3* in the supernatants were determined with ELISA. Results shown are representative of at least three independent experiments.

*IL-17* secretion in serum was significantly increased in patients with active SSc (35.9 ± 4.7, *n* = 13) compared with those with stable SSc (10.5 ± 2.6, *P* < 0.01, *n* = 32) and healthy controls (8.1 ± 2.0, *P* < 0.05, *n* = 24; Figure 4A). These data were consistent with a previous report that circulatory *IL-17* levels are increased in SSc patients [8]. We further showed that *IL-17* secretion from stimulated PBMCs of patients with active SSc was increased compared with PBMCs from patients with stable SSc and healthy controls (Figure 4B).

We found that *IL-17* alone could promote fibroblast growth as measured by MTT assay (See Additional file 1: Figure S1A). Furthermore, *IL-17* could induce *collagen 1* and *collagen 3* mRNA expression in fibroblasts in a dose-dependent manner (See Additional file 1: Figure S1B). These data indicated that *IL-17* could induce fibroblast growth and collagen production. To determine further whether *IL-17* derived from patients with active SSc can induce fibroblast growth and collagen production, we prepared supernatants from stimulated PBMCs of patients with active SSc in culture, and investigated its effect on the expression of *collagen 1* and *collagen 3* in fibroblasts. We found that culture supernatants from PBMCs of patients with active SSc promoted both mRNA expression and protein secretion of *collagen 1* and *collagen 3* in fibroblasts (Figure 4C, D). More notably, neutralization of *IL-17* in the culture medium inhibited mRNA expression and protein secretion of *collagen 1* and *collagen 3* (Figure 4C, D). Furthermore, our data showed that supernatants from stimulated PBMCs of active SSc patients could dose- and time-dependently induce the *collagen 1* and *collagen 3* mRNA (See Additional file 2: Figure S2). These data indicate that fibroblasts are responsive to stimulation by *IL-17* produced by PBMCs derived from SSc patients.

**Figure 5:**
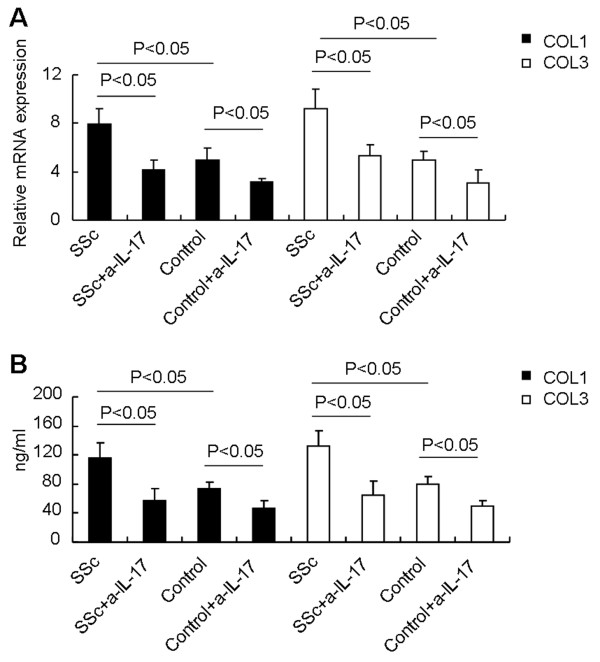
**Th17 cells contribute to fibroblast proliferation and collage production. (A)** Fibroblasts were stimulated for 48 hours with supernatants from cultures of PI-stimulated Th17 cells from patients with active SSc (Active) or healthy controls (Control) in the presence or absence of IL-17-neutralizing antibody, and the gene expression of *collagen 1* and *collagen 3* was analyzed with real-time RT-PCR analysis (*n* = 5 for each group). **(B)** Fibroblasts were stimulated for 48 hours with supernatants from cultures of PI-stimulated Th17 cells from patients with active SSc (Active) or healthy controls (Control) in the presence or absence of IL-17-neutralizing antibody, and the levels of *collagen 1* and *collagen 3* in the supernatants were determined with ELISA (*n* = 5 for each group).

Although *IL-17* derived from patients with active SSc could induce fibroblast growth and collagen production, it is not clear whether isolated Th17 cells have a similar effect. To determine whether Th17 cells from patients with active SSc induce collagen production in fibroblasts, CD4^+^CD161^+^CD196^+^ Th17 cells were sorted from PBMCs of SSc patients and healthy controls, and stimulated with PMA and ionomycin for 5 hours. The supernatants were collected and cocultured with fibroblasts. Our data showed that isolated Th17 cells from SSc patients produced more *IL-17* than that of healthy controls (data not shown). Furthermore, we showed that supernatants from Th17 cells of patients with active SSc induced more *collagen 1* and *collagen 3* production in fibroblasts than did supernatants of Th17 cells from healthy controls (Figure 5A, B), and neutralization of *IL-17* in the culture medium inhibited mRNA expression and protein secretion of *collagen 1* and *collagen 3*. Together, these data show that Th17 cell-derived *IL-17* from SSc patients could promote fibroblast growth and collagen production.

In addition, *IL-17* not only induced collagen secretion, but our data also showed that *IL-17* could promote the collagen synthesis (See Additional file 3: Figure S3A). Further data showed that *IL-17* could promote ERK phosphorylation in fibroblast, and ERK phosphorylation inhibitor could block IL-17-iduced fibroblast proliferation and collagen production (See Additional file 3: Figure S3B, C). These data suggest that the ERK signal pathway might be involved in the IL-17-mediated fibrosis in SSc patients.

## Discussion

The pathologic hallmark of SSc is excessive collagen deposition and microvascular injury. However, the mechanisms that lead to these changes remain largely unknown. An early skin mononuclear cell infiltrate consisting primarily of T cells and macrophages has been demonstrated [28]. Moreover, the degree of mononuclear cell infiltration in the skin of patients with SSc has been shown to correlate well with both the degree and progression of skin thickening [28]. Several lines of evidence suggest that T cells are important in the pathogenesis of SSc: first, T cells infiltrate skin early, before any evidence of fibrosis; second, an increased number of activated T cells is found in blood and skin lesions; third, T cells producing cytokines can induce fibroblast collagen production; fourth, T cells are necessary for antibody production; and fifth, treatments directed against T cells ameliorate systemic sclerosis [3]. These results bring the role of T cells in the pathogenesis of SSc to the forefront of the various mechanisms that may contribute to the pathogenesis of the disease. Although the role of immune dysfunction in the pathogenesis of SSc is generally accepted and strong evidence exists for the participation of T cells in the pathogenesis of this disease, the traditional Th1/Th2 paradigm has not been very helpful in explaining several aspects of the illness.

In our study, we showed that patients with active SSc had increased levels of circulating Th17 cells. In keeping with these observations, Th17 cell–derived *IL-17* was significantly higher in the serum of SSc patients compared with controls. In addition, increased infiltration of IL-17^+^ cells was present in involved skin of patients with early SSc. These data imply that Th17 cells are globally expanded in patients with active SSc rather than being redistributed. Although Th17 cells have been reported to account for several autoimmune diseases [29-31], the role of these cells in the course of fibrosis of SSc is not clearly understood. Our data showed that *IL-17* alone could induce fibroblast growth and collagen gene expression and protein secretion, *IL-17* derived from PBMCs and Th17 cells of patients with active SSc could promote collagen gene expression and protein production in fibroblasts, and neutralization of *IL-17in vitro* could block collagen production. Furthermore, Th17 cells isolated from active SSc could promote fibroblast growth and collagen production. *IL-17* not only induced collagen secretion, but promote fibroblast proliferation and the collagen synthesis (See Additional file 3: Figure S3). These data indicate that Th17 cell-derived *IL-17* may be involved in the fibrosis of SSc patients.

Treg cells are critical in maintaining self-tolerance and preventing autoimmunity [13,14] and have been implicated in the pathogenesis of several autoimmune diseases [32]. Our previous study also showed that Treg cells were depleted in patients with active SLE, which might be related to the expansion of Th17 cells [12]. In SSc patients, some reports have shown that although the number of Treg cells is markedly increased [4,33], these Treg cells have a diminished capacity to control CD4 effector T cells [34]. Our study showed that the number of circulating Treg cells decreased slightly, but not significantly, in patients with active SSc, which is partially consistent with previous findings that the percentage of Treg cells is decreased in SSc patients [21]. Treg cells dynamically change with the development of disease activity, and the enrolment of SSc patients with different disease activities might contribute to the discrepancy in the percentage of Treg cells among different studies. A major limitation of previous studies was that they did not determine whether Treg cells infiltrated the skin of patients with different stage of SSc, and the numbers of Treg cells that localized with skin inflammation was not clear. Our study showed that Foxp3^+^ Treg cells could be detected more frequently in both the epidermis and dermis of patients with early SSc compared with patients with stable SSc and healthy controls. Our unpublished data showed that the isolated circulating Treg cells did not affect fibroblast growth and collagen production. The upregulation of Foxp3^+^ cells in the skin of patients with early SSc may reflect a regulatory feedback mechanism to restore cellular tolerance and ameliorate harmful autoimmune responses.

One of the strengths of this study is the ability to analyze inflammatory cell subsets in involved skin of SSc patients with different clinical stages of disease. This enabled us to evaluate which complex inflammatory cell groups might be dynamically involved in the pathogenesis of SSc. Our data showed that Th17 cells were globally expanded in patients with active SSc and that Th17 cell-derived *IL-17* might be related to the fibrosis of SSc. Further studies into the role of Th17 cells and *IL-17* in fibrosis, as well as their effects in affected cells and tissue, are warranted. Furthermore, Th17 cell are only one of the factors for the fibrosis in SSc; more study should be done to make clear other lymphocytes or cytokines in the pathogenesis of fibrosis of SSc.

## Conclusions

Taken together, our data suggest that the pathophysiology of SSc might be linked to the expansion of Th17 cells and that Th17-derived *IL-17* may play a key role in the fibrotic course of SSc. These insights open novel avenues for research aimed at identifying pathogenic pathways and therapeutic targets.

## Abbreviations

DMEM: Dulbecco modified Eagle medium; dSSc: diffuse cutaneous SSc; ELISA: enzyme-linked immunosorbent assay; FITC: fluorescein isothiocyanate; IL-17: interleukin-17; lSSc: limited cutaneous SSc; MTT: 3-(4,5-dimethylthiazol-2-yl)-2,5-diphenyltetrazolium bromide; PBMCs: peripheral blood mononuclear cells; PBS: phosphate-buffered saline; PE: phycoerythrin; PMA: phorbol 12-myristate 13-acetate; RT-PCR: reverse transcription-polymerase chain reaction; SSc: systemic sclerosis; Th17: T helper 17 cells; Treg: T-regulatory cells.

## Competing interests

All authors declare that they have no competing interests.

## Authors’ contributions

ML had full access to all of the data in the study and takes responsibility for the integrity of the data and accuracy of the data analysis. Study design: XQY, JY, LLW, and ML. Acquisition of data: JY, XJX, LLW, and ML. Analysis and interpretation of data, XQY, JY, and ML. Manuscript preparation: JY and ML. Statistical analysis: XQY, JY, XJX, LLW, and ML. All authors read and approved the final version of the manuscript.

## Supplementary Material

Additional file 1: Figure S1 *IL-17* induces fibroblast proliferation and collagen production. **(A)** Fibroblasts isolated from SSc patients were cultured in 20 ng/ml *IL-17* or vehicle for the indicated number of days, and their growth was analyzed by MMT assay. **(B)** Fibroblasts were cultured in the indicated doses of *IL-17* for 48 hours; the gene expression of *collagen 1* and *collagen 3* was measured by real-time RT-PCR analysis. Click here for file

Additional file 2: Figure S2 *IL-17* derived from SSc patients induces collagen gene expression in fibroblasts. **(A)** Fibroblasts were stimulated with different concentrations of supernatants of PI-stimulated PBMCs from patients with active SSc (Active) and PI-stimulated PBMCs from healthy controls (Control) for 48 hours, and the gene expression of *collagen 1* and *collagen 3* was measured by real-time RT-PCR analysis. **(B)** Fibroblasts were stimulated with supernatants of PI-stimulated PBMCs from patients with active SSc (Active) and PI-stimulated PBMCs from healthy controls (Control) for different hours, and the gene expression of *collagen 1* and *collagen 3* was measured by real-time RT-PCR analysis. Results shown are representative of at least three independent experiments. Click here for file

Additional file 3: Figure S3 ERK activation is involved in the IL-17-mediated fibroblast proliferation and collagen production. **(A)** Fibroblast was stimulated with indicated doses of *IL-17* in the presence or absence of *IL-17* neutralizing antibody for 24 hours; collagen-1 and collagen-3 protein expressions in fibroblast were analyzed with Western blot. **(B)** Fibroblast was stimulated with *IL-17* and different doses of ERK- specific phosphorylation inhibitor-PD98059 for 24 hours; collagen-1 and collagen-3 protein expressions in fibroblast were analyzed with Western blot. **(C)** Fibroblast was stimulated with *IL-17* and ERK specific phosphorylation inhibitor-PD98059 for indicated days, the proliferation of fibroblast was examined by cell counting kit-8. Click here for file
