# Supplementary figures and images for: Increased frequency of Th17 cells in systemic sclerosis is related to disease activity and collagen overproduction
Source: Arthritis Res Ther. 2014 Jan 7;16(1):R4. doi: 10.1186/ar4430 (PMC3979142; doi:10.1186/ar4430)

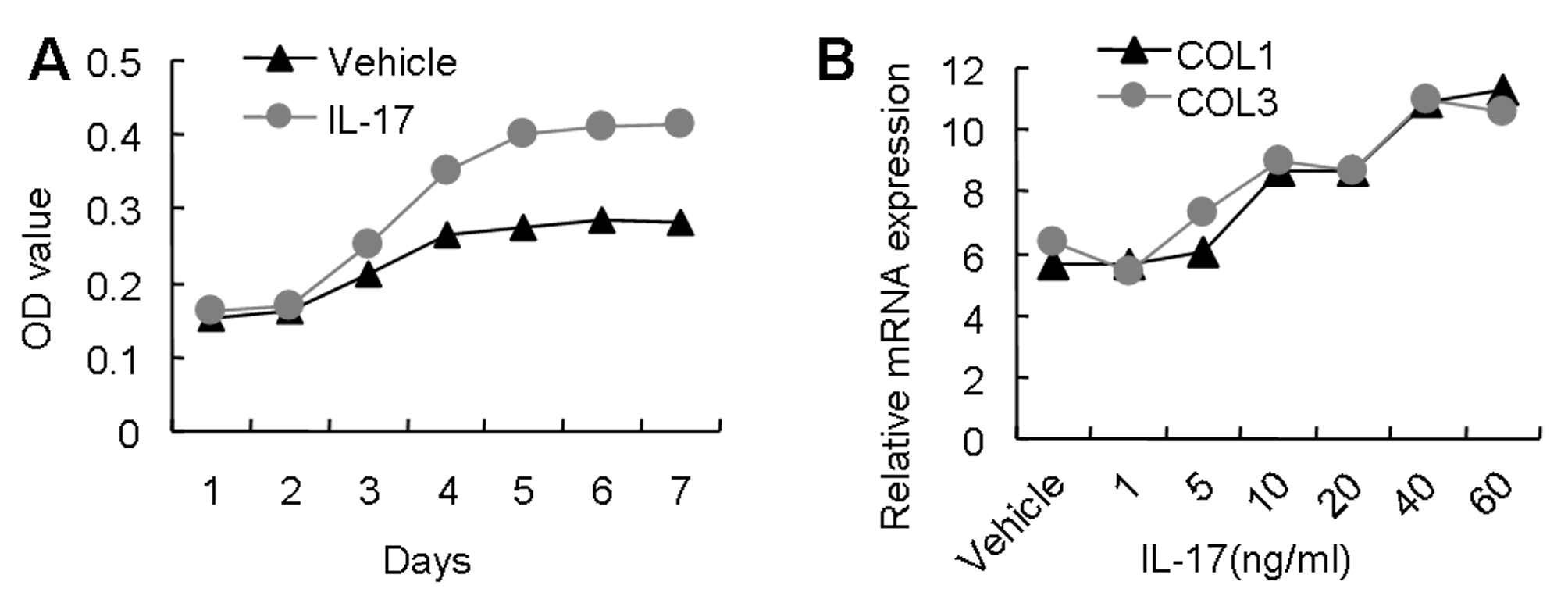

Supplement: Additional file 1: Figure S1 — IL-17 induces fibroblast proliferation and collagen production. (A) Fibroblasts isolated from SSc patients were cultured in 20 ng/ml IL-17 or vehicle for the indicated number of days, and their growth was analyzed by MMT assay. (B) Fibroblasts were cultured in the indicated doses of IL-17 for 48 hours; the gene expression of collagen 1 and collagen 3 was measured by real-time RT-PCR analysis. [file ar4430-S1.tiff]

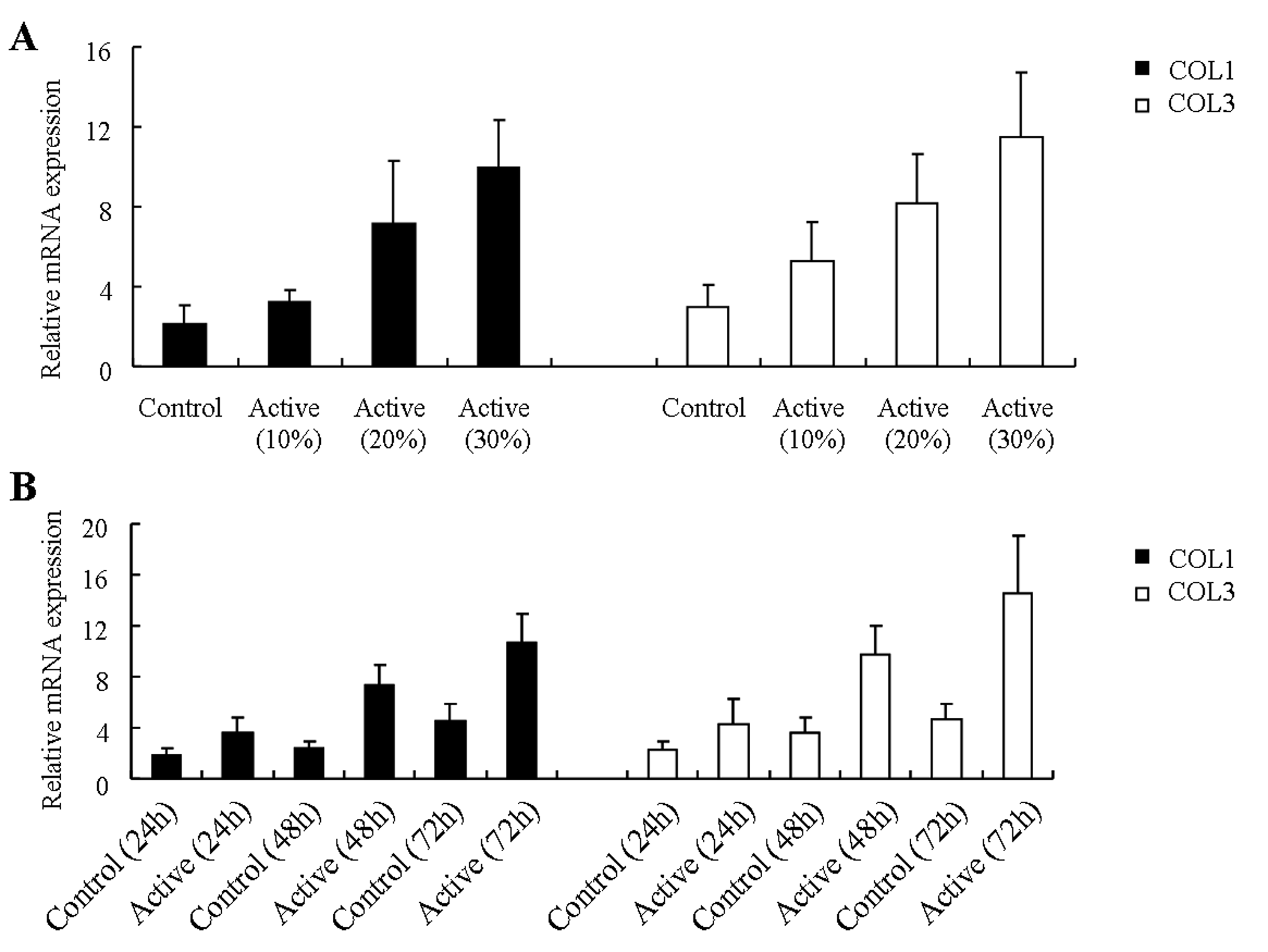

Supplement: Additional file 2: Figure S2 — IL-17 derived from SSc patients induces collagen gene expression in fibroblasts. (A) Fibroblasts were stimulated with different concentrations of supernatants of PI-stimulated PBMCs from patients with active SSc (Active) and PI-stimulated PBMCs from healthy controls (Control) for 48 hours, and the gene expression of collagen 1 and collagen 3 was measured by real-time RT-PCR analysis. (B) Fibroblasts were stimulated with supernatants of PI-stimulated PBMCs from patients with active SSc (Active) and PI-stimulated PBMCs from healthy controls (Control) for different hours, and the gene expression of collagen 1 and collagen 3 was measured by real-time RT-PCR analysis. Results shown are representative of at least three independent experiments. [file ar4430-S2.tiff]

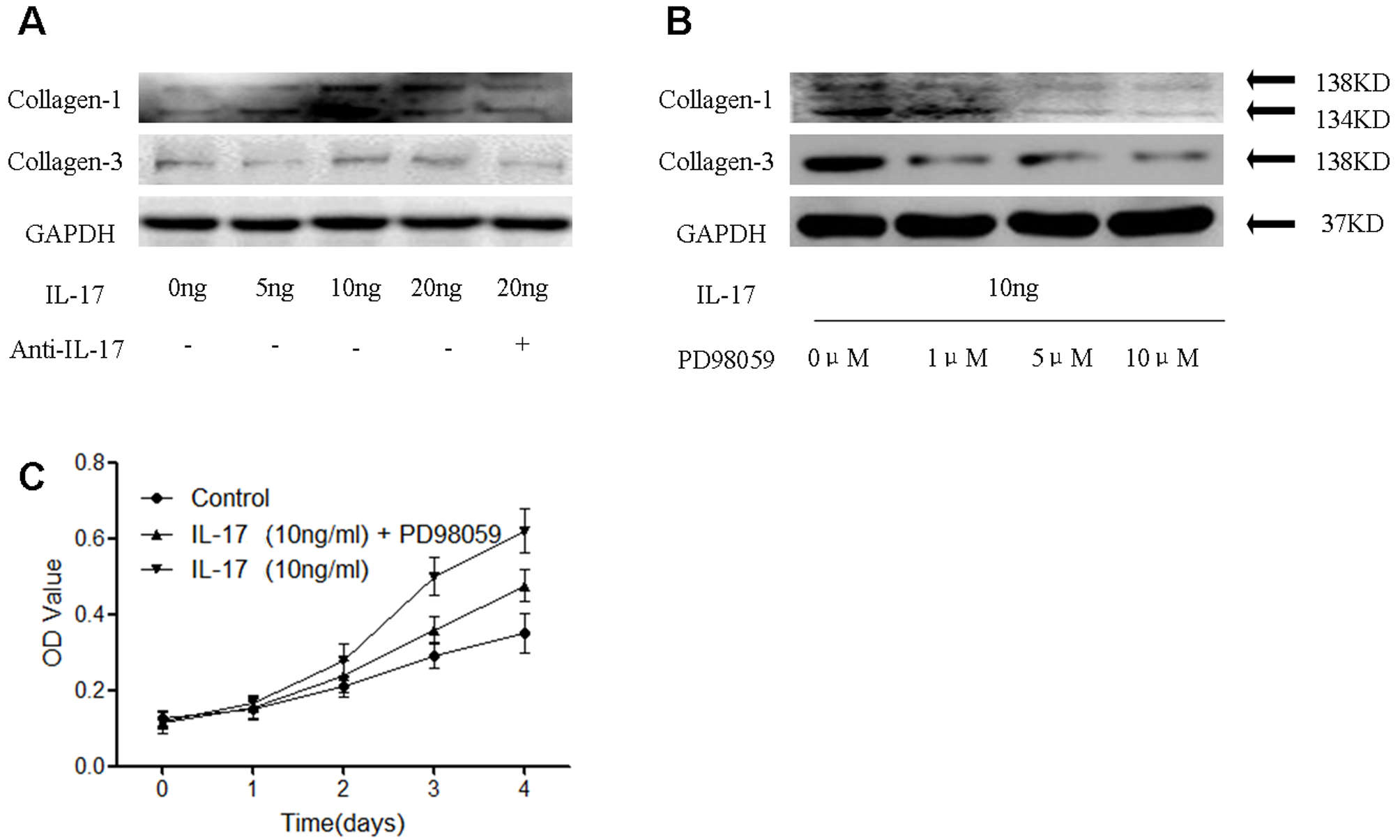

Supplement: Additional file 3: Figure S3 — ERK activation is involved in the IL-17-mediated fibroblast proliferation and collagen production. (A) Fibroblast was stimulated with indicated doses of IL-17 in the presence or absence of IL-17 neutralizing antibody for 24 hours; collagen-1 and collagen-3 protein expressions in fibroblast were analyzed with Western blot. (B) Fibroblast was stimulated with IL-17 and different doses of ERK- specific phosphorylation inhibitor-PD98059 for 24 hours; collagen-1 and collagen-3 protein expressions in fibroblast were analyzed with Western blot. (C) Fibroblast was stimulated with IL-17 and ERK specific phosphorylation inhibitor-PD98059 for indicated days, the proliferation of fibroblast was examined by cell counting kit-8. [file ar4430-S3.tiff]
